# Functional determination of site-mutations in *rdxA* involved in metronidazole resistance of *Helicobacter pylori*

**DOI:** 10.3389/fcell.2024.1435064

**Published:** 2024-07-19

**Authors:** Jia Huang, Zhiyu Li, Fulin Ge, Chao Sun, Zixin Deng, Weiyan Yao, Xinyi He

**Affiliations:** ^1^ Department of Gastroenterology, Ruijin Hospital Affiliated to Shanghai Jiao Tong University School of Medicine, Shanghai, China; ^2^ State Key Laboratory of Microbial Metabolism, Joint International Research Laboratory of Metabolic and Developmental Sciences, School of Life Sciences and Biotechnology, Shanghai Jiao Tong University, Shanghai, China

**Keywords:** RdxA site-mutation, Metronidazole resistance, *Helicobacter pylori*, Hp infection in Shanghai, Nucleic acid SNP detection

## Abstract

**Background:**

Metronidazole (MTZ) is among the first-line drugs against the human gastric pathogen *Helicobacter pylori* (*H. pylori*). MTZ is used as a prodrug that is activated by an oxygen-insensitive enzyme NADPH nitroreductase (RdxA). Loss-of-function mutations in *rdxA* make *H. pylori* MTZ resistant; however, experimental proof is lacking.

**Methods:**

We collected 139 gastric biopsy samples from patients suspected of *H. pylori* infection in Shanghai, and amplified *Hp*-specific *rdxA* gene from 134 samples. All these *rdxA* genes were sequenced and phylogenetically compared. The effect of mutations on RdxA function was measured by expressing them in *Escherichia coli* DH5α by using the MTZ sensitivity test.

**Results:**

In total, 134 gastric biopsy samples were identified as *H. pylori* positive. Of the 134 samples, 74 and 6 had point mutations at the various sites or promoter region of *rdxA*, generating truncated and extended fused proteins, respectively. The remaining 54 were full-length with single nucleotide variation (SNV) compared with the wild-type RdxA from *H. pylori*, with 49 clustering with hpEastAsia, 3 with hpEurope, and 2 with hpNEAfrica. All 134 *rdxA* were expressed in *E. coli* DH5α; 22 and 112 resultant strains showed MTZ-sensitive and MTZ-resistant phenotypes, respectively. Comparative analysis of single nucleotide polymorphisms (SNPs) in the functional and inactivated RdxA revealed 14 novel mutations in RdxA, 5 of which conferred MTZ resistance: S18F, D59S, L62I, S79N, and A187V.

**Conclusion:**

The occurrence of MTZ resistance induced by site-mutation of RdxA in patients with *H. pylori* infection was 83.6% (112/134) in the Shanghai region. The major form of loss-of-function mutation was truncation of RdxA translation at a rate of 58/112 (51.8%). Molecular detection reliably determined the resistance of *H. pylori* to MTZ. Thus, the functional mutants involved in MTZ resistance facilitate clinical diagnosis and medication based on sequence analysis.

## Introduction

*Helicobacter pylori* (*H. pylori*) is a gram-negative microaerophilic bacterium that was first isolated from the gastric antrum and cultivated *in vitro* (Marshall and Warren, 1984). In 1994, the World Health Organization classified *H. pylori* as a type-1 carcinogen (IARC, 1994). *H. pylori* infection is the major causative factor of peptic ulcer and chronic gastritis, affecting >50% of the world’s population. The average infection rate of *H. pylori* in different regions of China is ∼50% (Hooi et al., 2017). Because of the pathogenic and harmful nature of *H. pylori*, almost all patients with *H. pylori* infection require eradication therapy.

Treating *H. pylori* infection in a clinical setting is a huge challenge for physicians, and choosing the right antibiotic is critical for recovery from *H. pylori* infection (Savoldi et al., 2018). Traditional triple therapy, consisting of a proton pump inhibitor and two antibiotics, or quadruple therapy, consisting of a proton pump inhibitor, bismuth, and two antibiotics, is the most commonly used regimen (Kate et al., 2013).

Metronidazole (MTZ), a synthetic 5-nitroimidazole derivative of azomycin (Dingsdag and Hunter, 2018), is one of the constituents of standard triple therapy recommended by the National Institute of Health Consensus, 1994, for *H. pylori* eradication (NIH, 1994). In *H. pylori*, MTZ is activated by RdxA (Tomb et al., 1997) through the transfer of four electrons, which may produce intermediates, such as superoxide radicals, nitroso derivatives, and hydroxylamine, leading to DNA damage-mediated killing of *H. pylori* (Lindmark and Müller, 1976; Hu et al., 2017). This process is particularly active in anaerobic bacteria through the redox of pyruvate-ferredoxin oxidoreductase (PFOR) in concert with ferredoxin or effectors with a sufficiently negative midpoint redox potential (<−415 mV) (Lockerby et al., 1984). MTZ is used as an alternative in patients allergic or resistant to clarithromycin or amoxicillin (Gisbert and Pajares, 2005). However, MTZ, as a common antibiotic for anaerobic bacteria, is widely used in parasitic infections, gingivitis and vaginosis (Löfmark et al., 2010). Due to its mutation-inducing properties, its resistance is higher than that of clarithromycin and amoxicillin in developing countries, posing a great challenge for subsequent eradication of *H. pylori* as triple therapy (Savoldi et al., 2018). Therefore, it is necessary to perform a sensitivity test before usage.

Mutations in *rdxA*, which encodes an oxygen-insensitive NADPH nitroreductase, are the main causes of *H. pylori* resistance to MTZ (Goodwin et al., 1998). RdxA is a homodimer, which exhibits potent NADPH oxidase activity under aerobic conditions and MTZ reductase activity under anaerobic conditions with flavin mononucleotide (FMN) as cofactor (Olekhnovich et al., 2009). RdxA contains six cysteine residues with a pI of *ca*. 8, whereas the nitrogen regulators of enteric bacteria have 1–2 cysteine residues and more acidic pI values (pI 5.4–5.6). The C159 mutation leads to a significant decrease in MTZ reductase activity, indicating that the point mutations affect RdxA activity (Goodwin et al., 1998; Martínez-Júlvez et al., 2012).

Molecular testing methods, including restriction fragment length polymorphism (Suzuki et al., 2013), fluorescence *in situ* hybridization (Hansomburana et al., 2012), real-time polymerase chain reaction (RT-PCR) (Monno et al., 2012), and allele-specific-PCR (Nishizawa et al., 2007), and dual-priming oligonucleotide-based multiplex PCR (Woo et al., 2009), have been used to determine *H. pylori* resistance. The most commonly used target genes for the molecular detection of *H. pylori* include 23S *rRNA* and *gyrA*, because >90% of clarithromycin resistance is caused by mutations in A2142 and A2143 of *23S rRNA*, whereas quinolone resistance is mainly caused by mutations in the quinolone resistance determination region of *gyrA* (Gong and Yuan, 2018). By comparison, mutations in *rdxA* are variable and random, and some are developmental signals rather than resistance related (Zhang et al., 2020). This greatly increases the limitations of direct determination of MTZ resistance by molecular testing. However, it is difficult to perform MTZ susceptibility test in most clinical laboratories because the cultivation of *H. pylori* is challenging and time-consuming, with a low success rate.

In this study, we employed heterologous expression of *rdxA* in *E. coli* DH5α deficient in DNA repair to establish the relationship between site mutations of *rdxA* and MTZ resistance. The sequence map for MTZ resistance-related mutations from Shanghai is presented. In combination with multiple sequence analysis, we determined five new resistance-associated mutation sites in *rdxA*, facilitating clinical diagnosis and medication, based only on sequence analysis.

## Materials and methods

### Gastric biopsy sample

Participants (age >18  years) with positive ^13^C urea breath test from Ruijin Hospital, Shanghai Jiao Tong University were invited to participate. Exclusion criteria included (i) use of proton pump inhibitors, H_2_-receptor antagonists, or antibiotics within the previous 30 d; (ii) prior gastrectomy; (iii) history of severe heart, liver, and renal disorders; and (iv) pregnancy or breastfeeding. The study protocol was approved by the Ethics Committee of Ruijin Hospital (Ethics Approval Number: (2022)321), and written informed consent was obtained from all participants. Gastric mucosa biopsy samples were obtained during upper endoscopy between January 2022 and December 2022. Two pieces of tissue, collected from the antrum 2–3 cm in front of the pylorus, were used for PCR detection and pathological examination.

### Rapid preparation of total DNA from *H. pylori*

DNA was extracted from biopsies using carboxy magnetic beads (Tiangen Biotech, China). Briefly, the samples were incubated with 600 μL of lysis buffer (2% SDS) for 5 min at 95°C; mixed with 600 μL of isopropyl alcohol and vigorously vortexed for 5  s; cooled to room temperature; and mixed and incubated with 10 μL of magnetic beads (10 mg/mL) for 5 min at room temperature. The supernatant was discarded, and the magnetic beads with absorbed nucleic acid were collected using a Magnetic Separation Device (Sangon Biotech, China). The magnetic beads were washed twice with 80% ethanol and incubated for 5 min with 200 μL of RNase-free water to elute nucleic acid. The supernatant was transferred to a new tube and stored at −40°C. A simple flowchart for DNA extraction is shown in Supplementary Figure S1A.

### PCR detection of *H. pylori*-specific genes

The sequences of 23S *rRNA*, *oorD*, *rdxA*, *frxA*, and 16S *rRNA* available for *H. pylori* strains were downloaded from the NCBI database to obtain conserved regions. The conserved fragments were PCR amplified using total DNA from the samples (all primers listed in Supplementary Table S1). The concentration of each primer was 200 nM. Primer specificity was verified by Primer-BLAST. PrimeSTAR Max DNA Polymerase was used for singleplex PCR and Ex Taq DNA Polymerase for multiplex PCR (TaKaRa). DNA quality was acceptable for PCR amplification of *H. pylori 23S rRNA* (Supplementary Figure S1B) and multiplex amplification of *H. pylori* 23S *rRNA*, *oorD*, *rdxA*, *frxA*, and 16S *rRNA* (Supplementary Figure S1C).

### Construction of the *rdxA* expression vector and site-directed mutagenesis

*rdxA* obtained from total DNA was amplified by PCR using the primers *rdxA*-F1 and *rdxA*-R1. The resulting amplicons were 860 bp in length. The product was then amplified using the primers *rdxA*-F2 and *rdxA*-R2, with restriction sites *EcoR*I and *BamH*I, respectively. The amplified product and pBluescript were digested with the same restriction enzymes and ligated using DNA Ligation Kit Ver.2.1 (Takara). RdxA mutant variants were constructed using whole-plasmid PCR and *Dpn*I digestion. Primers used are listed in Supplementary Table S1. The sequence of *rdxA* was confirmed (Majorbio Co., Ltd., Shanghai). Confirmed sequences were transformed into *E. coli* DH5α (Invitrogen) for sensitivity testing.

### Construction of the phylogenetic tree

The phylogenetic tree was constructed using MEGA-X. The reference protein sequences compared were obtained from a broad range of geographic locations and contained seven population types (Supplementary Table S2). All aligned with full-length RdxA from samples using the Clustal W algorithm. The neighbor-joining method was employed to construct a phylogenetic tree with default values in MEGA-X. The phylogenetic tree was visualized on iTOL (iTOL: Interactive Tree Of Life (embl.de)).

### High performance liquid chromatography (HPLC) detection

The *E. coli* DH5α containing pBluescript *rdxA*-26695 or pBlueScript were cultured aerobically till an optical density of 0.6 was achieved at 600 nm in LB broth; mixed with MTZ to a final concentration of 16 μg/mL; and cultured overnight under anaerobic conditions. The supernatant was collected after centrifugation for HPLC detection using a YMC ODS-AQ chromatographic column. Solution A contained 0.5% (v/v) trifluoroacetic acid in water, whereas solution B was 100% acetonitrile. The ratios of solution A to B (v/v) in mobile phases A and B were (5:95) and (90:10), respectively. The flow rate, wavelength for determination, and temperature of the column were 1 mL/min, 320 nm, and 25°C, respectively.

### Sensitivity test

*E*. *coli* DH5α expressing *rdxA* from samples and *H. pylori* 26695 were cultured aerobically to OD_600_ 0.5 in LB broth and spotted (5 μL of each strain) onto LB agar plates supplemented with 0–16 μg of MTZ (Sigma-Aldrich) per mL. The plates were incubated under aerobic conditions at 37°C and then scored for growth at 16–24 h.

### Data analysis

Correlation analysis was performed by R. The effect of mutations was investigated based on the crystal structure of *H. pylori* RdxA strain 26695 [Protein Data Bank (PDB): 3QDL]. Residue exchange was modeled and analyzed with PyMol. The energetic effect of the mutation was quantified using DUETweb.

## Results

### Baseline characteristics and good consistency between clinical and molecular diagnosis

A total of 139 clinical samples were obtained from patients with positive ^13^C urea breath tests at Shanghai Ruijin Hospital. Overall, *H. pylori* positive results were concordant between pathological examination and PCR detection in 134 patients, and one sample was negative when assessed using either method. In one case, *H. pylori* was identified in pathological examination but not in PCR detection, and in three cases *H. pylori* was detected only by PCR. Clinical data showed that of the 134 patients with *H. pylori* infection and investigated by gastric endoscopy, 98 (73.13%) had gastritis, 10 (7.46%) had gastric ulcer, and 26 (19.41%) had duodenal ulcer (Table 1).

**TABLE 1:** Demographic characteristics and diagnosis of the recruited patients with positive *H. pylori* infection (n = 134).

Baseline characteristics (n = 134)	
Age, Years (mean ± SD)	47.54 ± 13.25
Gender (%)	
Male	63 (47.01%)
Female	71 (52.99%)
Gastric lesions (%)	
Gastritis	98 (73.13%)
Gastric ulcer	10 (7.46%)
Duodenal ulcer	26 (19.41%)

### Mutation analysis of *rdxA* of *H. pylori* from clinical samples

*H. pylori* RdxA is responsible for susceptibility to the redox active prodrug MTZ (Goodwin et al., 1998). Therefore, *rdxA* from 134 clinical samples positive for *H. pylori* were cloned and sequenced to identify mutations associated with resistance phenotypes. Compared to the reference RdxA from *H. pylori* 26695, 74 and 6 had point mutations at the various sites or promoter region of *rdxA*, generating truncated and extended fused protein respectively, whereas 54 were full-length with missense point mutations. Of the 74 truncated variants, 27 were pretermination, 33 were deletions and 14 were insertions. The 6 fusion variants were promoter deletion, in which the start codon M was mutated to I (Table 2).

**TABLE 2:** Statistics of mutations on RdxA.

	Mutation type	Clinical samples (ratio%)
Full-length	Missense mutation	54 (40.3)
Truncation	Pre-termination	27 (20.15)
	Deletion	33 (24.63)
	Insertion	14 (10.45)
Fusion	Promoter deletion	6 (4.47)

**FIGURE 1:**
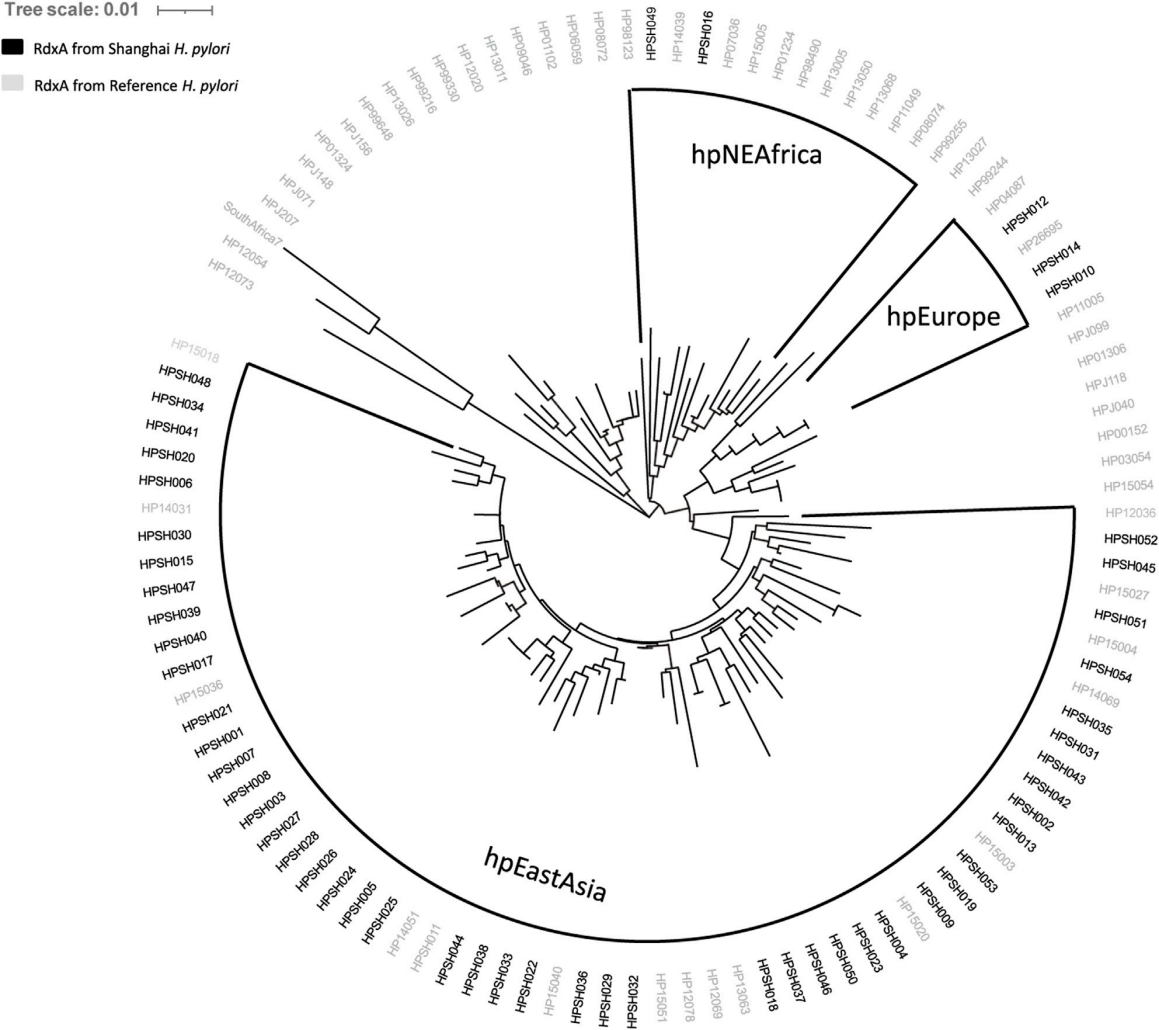
Cluster analysis of full-length RdxA sequence from Shanghai samples. Comparison of the sequence homology of RdxA from samples from Shanghai with 57 typical sequences belonging to the seven major population clusters. Populations containing sample sequences are highlighted with shading. Most sample RdxA sequences are clustered with the East Asian group.

According to the characteristics of geographical distribution (Falush et al., 2003; Lamichhane et al., 2020), *H. pylori* have been divided into seven distinct populations: hpEurope, hpEastAsia, hpAfrica1, hpAfrica2, hpAsia2, hpNEAfrica, and hpSahul. The reference strain *H. pylori* 26695 used in this study belongs to hpEurope; 57 RdxA sequences of representative strains from different populations were aligned with 54 full-length RdxA sequences (Supplementary Table S1) (Lauener et al., 2019). Among them, 49 samples clustered with hpEastAsia, 3 were hpEurope, and 2 were hpNEAfrica (Figure 1). Considering Shanghai as an international hub, the 5 strains from outside hpEastAsia may be associated with increased human mobility and large-scale international export of food products (Yang et al., 2021). Compared to the RdxA reference, mutations, such as T31E, H53R, D59N, L62V, S88P, G98S, R131K, and V172I (Table 3), were conservatively present in >68% of RdxA sequences. They exclusively occurred in hpEastAsia strains (Supplementary Table S3), suggesting that these residue shifts may be evolutionary traces other than resistance-related mutations.

**TABLE 3:** List of amino acid substitution sites detected in full-length RdxA.

Amino acid substitution	Clinical samples (n = 54) (%)
R131K	94.44
T31E, S88P, G98S	90.74
D59N, V172I	88.89
H53R	81.48
L62V	68.52
A206T	40.74
A118S	22.22
S108A, R16H, M21A	14.81
D5N, P91S	11.11
R16C, A68V, V204I	7.41
D59S, A80T, G163V, E174K	5.56
S116F, P180S, Q6H, H25R, A68T, S92N, H97Y, A193T	3.70
S43L, Y135S, S134N, S18F, M21I, H25Y, S30G, Y47C, L62I, M56I, K64N, E74G, S79N, A82V, R90K, P96L, H97T, A118T, Q146R, A187V, A193V, S29T	1.85

### Identification of new mutation sites in MTZ-resistant strains

Because the cultivation of *H. pylori in vitro* is challenging and time-consuming and has a low success rate, we examined the activity of RdxA variants by heterologous expression of their coding genes in *E. coli* DH5α. There are two reasons to choose this host: 1) it’s a DNA recombination-deficient strain that is susceptible to DNA damage (Supplementary Figure S2); and 2) it lacks a *rdxA* homolog, and is resistant to MTZ (Jackson et al., 1984). We individually cloned 134 *rdxA* genes with 102-bp upstream DNA sequences, into pBluescript and transformed these into *E. coli* DH5α for MTZ sensitivity testing. Compared with *E. coli* DH5α harboring *rdxA* from *H. pylori* 26695 (MIC ≤ 8 μg/mL), expression strains with 112 *rdxA* showed MTZ-resistant phenotypes (MIC > 8 μg/mL). Thus, in patients with *H. pylori* infection in Shanghai, MTZ resistance was 83.58%. For the 112 MTZ-resistant RdxA, 64 were truncations or fusions, and 48 were missense mutations with full-length coding sequences. Comparative analysis of RdxA from MTZ-susceptible and -resistant strains revealed that 11 mutations occurred exclusively in resistant strains: S18F, S30G, D59S, L62I, S79N, A82V, S92N, Q146R, E174K, A187V, and A193V. These SNPs have not been reported to confer MTZ resistance in *H. pylori*. Two new mutations occurred only in susceptible strains: S29T and S43L, and 1 new mutation P180S occurred in both susceptible and resistant strains (Table 4). The importance of these 14 sites identified from strains with resistant, susceptible and resistant/susceptible phenotype to MTZ were further functionally explored.

**TABLE 4:** Comparison of SNPs detected in the 54 full-length RdxA proteins.

Codon	Number of susceptible *E. coli* isolates	Number of resistant *E. coli* isolates	This study	Reference
S29T	1	—	S	—
S43L	1	—		—
S134N	1	—		R/S
Y135S	1	—		R/S
D5N	1	5	R/S	S
T31E	8	41		R/S
H53R	6	38		R/S
D59N	8	40		R/S
L62V	4	33		R/S
S88P	8	41		R/S
G98S	8	41		R/S
S108A	1	7		R/S
S116F	1	1		R
A118S	2	10		R/S
R131K	8	43		R/S
V172I	8	40		R/S
P180S	1	1		—
A206T	5	17		R
Q6H	—	2	R	R/S
R16H	—	8		R
R16C	—	4		R
S18F	—	1		—
M21A	—	8		R/S
M21I	—	1		R
H25Y	—	1		R
H25R	—	2		R
S30G	—	1		—
Y47C	—	1		R
D59S	—	3		—
L62I	—	1		—
M56I	—	1		R/S
K64N	—	1		R/S
A68V	—	4		R/S
A68T	—	2		R
E74G	—	1		R/S
S79N	—	1		—
A80T	—	3		R/S
A82V	—	1		—
R90K	—	1		R/S
P91S	—	6		R/S
S92N	—	2		—
P96L	—	1		S
H97Y	—	2		R/S
H97T	—	1		R
A118T	—	1		R/S
Q146R	—	1		—
G163V	—	3		R
E174K	—	3		—
A187V	—	1		—
A193T	—	2		R
A193V	—	1		—
V204I	—	4		R/S

“R” means the mutation has been reported to occur in metronidazole resistance *H. pylori* strains.

“S” means the mutation has been reported to occur in metronidazole susceptible *H. pylori* strains.

### Functional determination of site mutations involved in MTZ resistance

**FIGURE 2:**
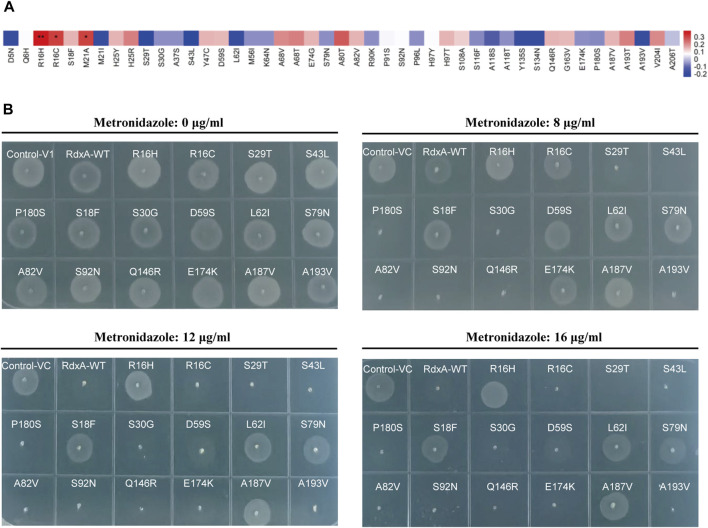
Association of RdxA mutations with MTZ resistance **(A)** Correlation coefficient between 45 mutations present in full-length RdxA and MTZ resistance was calculated as Spearman’s correlation (**P < 0.01; *P < 0.05). **(B)** The 16 mutation sites were randomly selected for verification, and their drug-resistant phenotypes were observed on LB plates containing 0, 8, 12, and 16 μg/mL MTZ.

To correlate the site mutations and MTZ resistance, mutations on the basis of the RdxA sequence from *H. pylori* 26695, including R16H, R16C (both as control), S29T, S43L, P180S, S18F, S30G, D59S, L62I, S79N, A82V, S92N, Q146R, E174K, A187V, and A193V, were made and expressed in DH5α. MIC values of MTZ resistance for these strains expressing RdxA variants (Table 4) were measured. Consistent with previous reports, R16H significantly correlated with increase in MTZ resistance (**P < 0.01) (Figure 2A) (Zhang et al., 2020). In addition, S18F, D59S, L62I, S79N, and A187V showed moderate enhancement in MTZ resistance (Figure 2B). These loci might be used as molecular diagnostic targets for MTZ sensitivity based on DNA sequence, and thus avoid unnecessary use of MTZ.

### Effect of point mutations on the structure and function of RdxA

**FIGURE 3:**
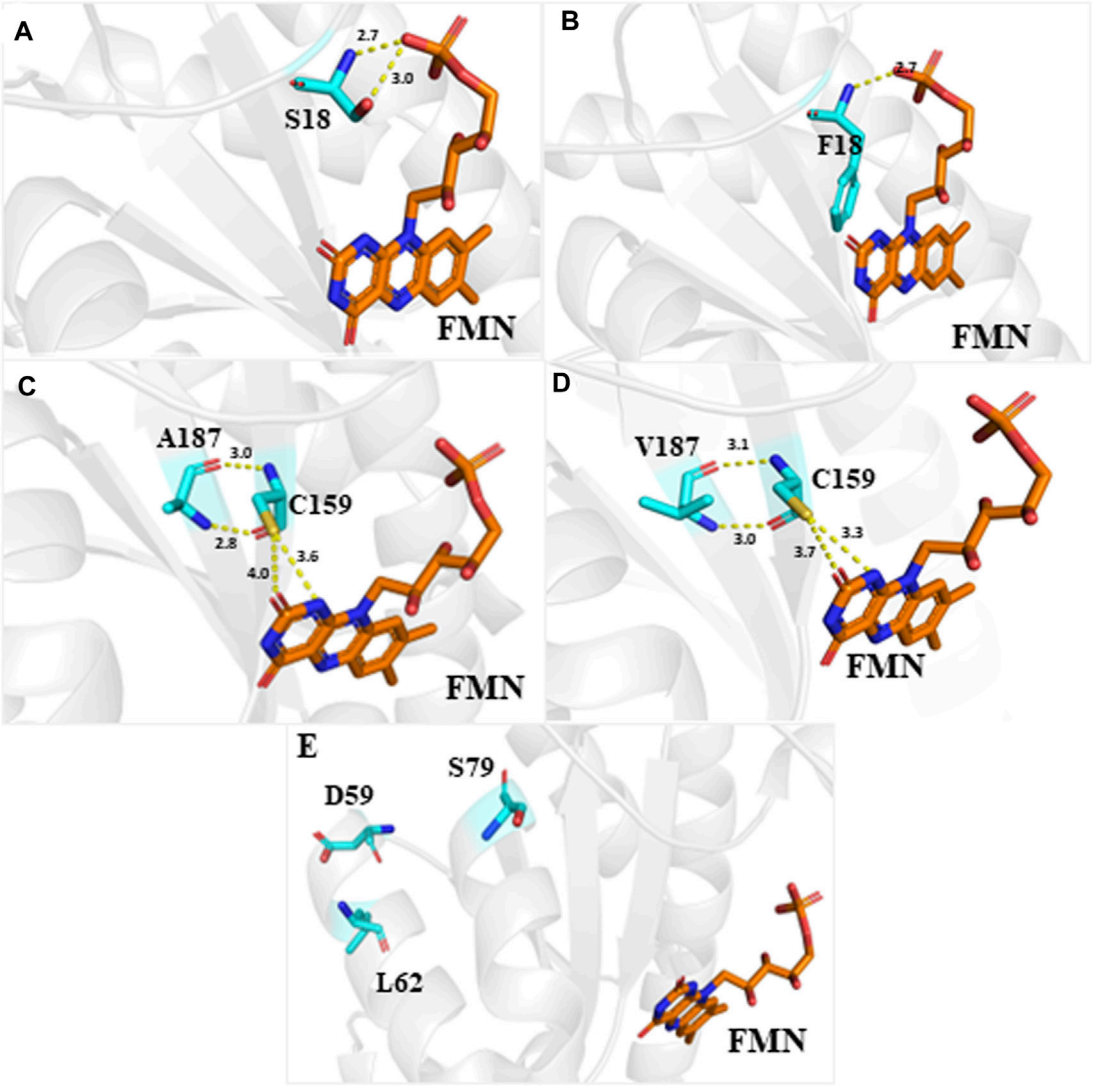
Crystal structure of dimeric *H. pylori* RdxA with the cofactor FMN Structure of RdxA is shown in gray. Mutation sites, including S18, D59, L62, S79, and A187, and the cofactor FMN are highlighted using different colors. **(A,B)** Position S18 was mutated to F18, resulting in a decrease of three hydrogen bond interactions between the amino acid and FMN to one. **(C,D)** Position A187 was mutated to V187, resulting in the loss of hydrogen bonding with C159, which interacts with FMN. **(E)** The left three mutation sites D59, L62, and S79 are located at α helix. Hydrogen bonds are indicated by yellow dashed lines.

We obtained the crystal structure of RdxA from PDB to investigate the SNPs important for RdxA activity. RdxA is a homodimer that exhibits domain swapping and contains two molecules of FMN bound at the dimer interface (Martínez-Júlvez et al., 2012). The positions of S18, D59, L62, S79, and A187 are mapped onto the RdxA structure (Figure 3). The hydrogen bonds formed by FMN ribi tyl and hydroxyl of Ser18 of the same monomer decreased when S18 was changed to F18 with the bond of 3.0 Å disappeared which may explain the decrease in RdxA activity (Figures 3A, B). The other four mutations were >8 Å from FMN and could not interact with the latter. Among them, N and O of A187 form hydrogen bonds with O and N of C159, which is important for MTZ reductase activity. A187V mutation slightly increased the lengths of two bonds with C159, which finally influenced the positioning of FMN (Figures 3C, D). Previous studies have shown that two functional homodimers are present in the asymmetric unit of the tetragonal crystals in the RdxA structure. Interestingly, the remaining three mutation sites, D59S, L62I and S79N, located on α helixs, which were away from the dimer interface and did not interact with cofactor FMN (Figure 3E) (Martínez-Júlvez et al., 2012). Considering the importance of α helixes in stability of protein conformation, mutations of them may destruct the primary structure of RdxA and influence its activity.

## Discussion

With the global increase in the prevalence of antibiotic resistance and poor availability of culture-based susceptibility testing, the empirical use of most popular triple therapies for *H. pylori* has resulted in poor cure rates (Graham and El-Serag, 2021; Nyssen et al., 2021). Studies have confirmed that quadruple therapy, including clarithromycin, levofloxacin, or MTZ, should only be used after confirmation of culture susceptibility (Graham and Moss, 2022). In China, antimicrobial resistance in *H. pylori* is especially prevalent, and molecular-based susceptibility testing is recommended to predict *H. pylori* resistance to antibiotics. Consistently, resistance to antibiotics, such as clarithromycin and levofloxacin, in *H. pylori* is well understood, and molecular-based tests could provide regional *H. pylori* susceptibility data to enhance cure rates. However, the molecular underpinnings of MTZ are controversial.

Gene sequencing alone cannot confirm whether *H. pylori* is resistant or susceptible to MTZ in a given patient because the exact site mutations that lead to the functional failure of RdxA remain unknown. Many spontaneous mutations that are not essential to RdxA function further complicate the determination of resistance-related sites simply based on gene sequence. RdxA can reduce MTZ in *E. coli* and cause DNA lesions. In most cases, these lesions can be repaired using a DNA recombination system, such as RecA. However, DNA lesions introduced by MTZ in RdxA are lethal to *E. coli* DH5α, which is deficient in DNA repair. This feature offered a good opportunity to correlate between MTZ resistance with site mutations in RdxA.

Failures in the eradication of *H. pylori* may be due to preexisting drug-resistant *H. pylori* strains or the development of uniquely resistant strains due to the antimicrobial burden, and point mutations are a major mechanism of antibiotic resistance (Binh et al., 2015). MTZ resistance is predominantly caused by mutations in RdxA (Goodwin et al., 1998). RdxA sequences are highly heterogeneous, with the homology of sensitive strains in clinical samples ranging 95.73%–96.21%. Our study included 134 patients with *H. pylori* infection, and mutations in full-length RdxA identified in 54 patients could be classified into three types: (i) evolutionary traces rather than drug resistance mutations, such as T31E, H53R, D59N, L62V, S88P, G98S, R131K, and V172I, which are conservatively present in some MTZ-sensitive strains belonging to a few phylogenetic clades (Supplementary Table S2); (ii) low-frequency random mutations not associated with drug resistance, accounting for 40.4% of the total mutation sites; and (iii) mutations occurring only in drug-resistant strains. For these sites, we analyzed the effect of mutations on the function of RdxA combined with its protein structure. R16H, S18F, and G163V were expected to reduce the affinity of the apo protein for FMN cofactors. D59S, L62I, and S79N were located in the α helix. These mutations may affect the structural stability of RdxA. Moreover, molecular docking study predicted 24 mutation sites related to MTZ resistance (Chu et al., 2020). M21A, M21I, and Y47C revealed in our study are among them.

In addition to full-length RdxA, the majority (44.78%) of RdxA sequences from clinical samples were truncated due to frameshift or nonsense mutations, most of which showed reduced activity. Interestingly, some strains expressing truncated RdxA exhibited an MTZ-sensitive phenotype. We found by sequence alignment that these proteins lacked the Q50–V55 or A68–E75 fragment, indicating that these fragments may not be critical for the nitro-reducing function of RdxA. Therefore, mutations in this region can be excluded from the MTZ resistance correlation analysis. Furthermore, 27/60 truncated sequences were due to a premature termination codon. Aminoglycoside antibiotics can restore full-length expression of truncated proteins through premature termination codon readthrough (Zingman et al., 2007). Gentamicin, netilmicin, and tobramycin have bactericidal effects on *H. pylori* (Lee et al., 2019). Therefore, the combination of MTZ and aminoglycoside antibiotics may have unexpected effects during *H. pylori* treatment. Interestingly, four MTZ-resistant frameshift mutations occurred upstream of *rdxA*, suggesting that the regulation of RdxA expression could result in MTZ resistance, consistent with previous reports (Han et al., 2007).

In conclusion, our study suggests that molecular detection can replace culture-based susceptibility testing for *H*. *pylori* to MTZ. The findings provide new resistance-associated mutation sites in *rdxA* to increase predictive accuracy, particularly for MTZ resistance. We believe that sequence analysis will reliably predict outcomes of *H. pylori* infection treatment in the future.

## Data Availability

The sequence data presented in the study are deposited in the GenBank, accession numbers (PP950248 and PP950301) for these sequences are listed in the first column of Supplementary Table S3.

## References

[B1] Binh T. T. Suzuki R. Trang T. T. Kwon D. H. Yamaoka Y. (2015). Search for novel candidate mutations for metronidazole resistance in *Helicobacter pylori* using next-generation sequencing. Antimicrob. Agents Chemother. 59, 2343–2348. 10.1128/aac.04852-14 25645832 PMC4356779

[B2] Chu A. Wang D. Guo Q. Lv Z. Yuan Y. Gong Y. (2020). Molecular detection of *H. pylori* antibiotic-resistant genes and molecular docking analysis. Faseb J. 34, 610–618. 10.1096/fj.201900774R 31914672

[B3] Dingsdag S. A. Hunter N. (2018). Metronidazole: an update on metabolism, structure-cytotoxicity and resistance mechanisms. J. Antimicrob. Chemother. 73, 265–279. 10.1093/jac/dkx351 29077920

[B4] Falush D. Wirth T. Linz B. Pritchard J. K. Stephens M. Kidd M. (2003). Traces of human migrations in *Helicobacter pylori* populations. Science 299, 1582–1585. 10.1126/science.1080857 12624269

[B5] Gisbert J. P. Pajares J. M. (2005). *Helicobacter pylori* “rescue” therapy after failure of two eradication treatments. Helicobacter 10, 363–372. 10.1111/j.1523-5378.2005.00324.x 16181345

[B6] Gong Y. Yuan Y. (2018). Resistance mechanisms of *Helicobacter pylori* and its dual target precise therapy. Crit. Rev. Microbiol. 44, 371–392. 10.1080/1040841x.2017.1418285 29293032

[B7] Goodwin A. Kersulyte D. Sisson G. Veldhuyzen Van Zanten S. J. Berg D. E. Hoffman P. S. (1998). Metronidazole resistance in *Helicobacter pylori* is due to null mutations in a gene (rdxA) that encodes an oxygen-insensitive NADPH nitroreductase. Mol. Microbiol. 28, 383–393. 10.1046/j.1365-2958.1998.00806.x 9622362

[B8] Graham D. Y. El-Serag H. B. (2021). European Registry on *Helicobacter pylori* management shows that gastroenterology has largely failed in its efforts to guide practitioners. Gut 70, 1–2. 10.1136/gutjnl-2020-322385 PMC10506726 32958543

[B9] Graham D. Y. Moss S. F. (2022). Antimicrobial susceptibility testing for *Helicobacter pylori* is now widely available: when, how, why. Am. J. Gastroenterol. 117, 524–528. 10.14309/ajg.0000000000001659 35081545 PMC8976707

[B10] Han F. Liu S. Ho B. Yan Z. Yan X. (2007). Alterations in rdxA and frxA genes and their upstream regions in metronidazole-resistant *Helicobacter pylori* isolates. Res. Microbiol. 158, 38–44. 10.1016/j.resmic.2006.10.001 17113269

[B11] Hansomburana P. Anantapanpong S. Sirinthornpunya S. Chuengyong K. Rojborwonwittaya J. (2012). Prevalence of single nucleotide mutation in clarithromycin resistant gene of *Helicobacter pylori*: a 32-months prospective study by using hybridization real time polymerase chain reaction. J. Med. Assoc. Thai 95 (Suppl. 3), S28–S35. 22619884

[B12] Hooi J. K. Y. Lai W. Y. Ng W. K. Suen M. M. Y. Underwood F. E. Tanyingoh D. (2017). Global prevalence of *Helicobacter pylori* infection: systematic review and meta-analysis. Gastroenterology 153, 420–429. 10.1053/j.gastro.2017.04.022 28456631

[B13] Hu Y. Zhu Y. Lu N. H. (2017). Novel and effective therapeutic regimens for *Helicobacter pylori* in an era of increasing antibiotic resistance. Front. Cell Infect. Microbiol. 7, 168. 10.3389/fcimb.2017.00168 28529929 PMC5418237

[B14] IARC (1994). Schistosomes, liver flukes and *Helicobacter pylori*. IARC working group on the evaluation of carcinogenic risks to humans. Lyon, 7-14 june 1994. IARC Monogr. Eval. Carcinog. Risks Hum. 61, 1–241. 7715068 PMC7681621

[B15] Jackson D. Salem A. Coombs G. H. (1984). The *in-vitro* activity of metronidazole against strains of *Escherichia coli* with impaired DNA repair systems. J. Antimicrob. Chemother. 13, 227–236. 10.1093/jac/13.3.227 6373714

[B16] Kate V. Kalayarasan R. Ananthakrishnan N. (2013). Sequential therapy versus standard triple-drug therapy for *Helicobacter pylori* eradication: a systematic review of recent evidence. Drugs 73, 815–824. 10.1007/s40265-013-0053-z 23625272

[B17] Lamichhane B. Wise M. J. Chua E. G. Marshall B. J. Tay C. Y. (2020). A novel taxon selection method, aimed at minimizing recombination, clarifies the discovery of a new sub-population of *Helicobacter pylori* from Australia. Evol. Appl. 13, 278–289. 10.1111/eva.12864 31993076 PMC6976958

[B18] Lauener F. N. Imkamp F. Lehours P. Buissonnière A. Benejat L. Zbinden R. (2019). Genetic determinants and prediction of antibiotic resistance phenotypes in *Helicobacter pylori* . J. Clin. Med. 8doi, 53. 10.3390/jcm8010053 PMC6351930 30621024

[B19] Lee K. H. Park S. Y. Jeong S. J. Jung D. H. Kim J. H. Jeong S. H. (2019). Can aminoglycosides Be used as a new treatment for *Helicobacter pylori*? *in vitro* activity of recently isolated *Helicobacter pylori* . Infect. Chemother. 51, 10–20. 10.3947/ic.2019.51.1.10 30941933 PMC6446016

[B20] Lindmark D. G. Müller M. (1976). Antitrichomonad action, mutagenicity, and reduction of metronidazole and other nitroimidazoles. Antimicrob. Agents Chemother. 10, 476–482. 10.1128/aac.10.3.476 791102 PMC429775

[B21] Lockerby D. L. Rabin H. R. Bryan L. E. Laishley E. J. (1984). Ferredoxin-linked reduction of metronidazole in Clostridium pasteurianum. Antimicrob. Agents Chemother. 26, 665–669. 10.1128/aac.26.5.665 6517554 PMC179990

[B22] Löfmark S. Edlund C. Nord C. E. (2010). Metronidazole is still the drug of choice for treatment of anaerobic infections. Clin. Infect. Dis. 50 (Suppl. 1), S16–S23. 10.1086/647939 20067388

[B23] Marshall B. J. Warren J. R. (1984). Unidentified curved bacilli in the stomach of patients with gastritis and peptic ulceration. Lancet 1, 1311–1315. 10.1016/s0140-6736(84)91816-6 6145023

[B24] Martínez-Júlvez M. Rojas A. L. Olekhnovich I. Espinosa Angarica V. Hoffman P. S. Sancho J. (2012). Structure of RdxA--an oxygen-insensitive nitroreductase essential for metronidazole activation in *Helicobacter pylori* . Febs J. 279, 4306–4317. 10.1111/febs.12020 23039228 PMC3504637

[B25] Monno R. Giorgio F. Carmine P. Soleo L. Cinquepalmi V. Ierardi E. (2012). *Helicobacter pylori* clarithromycin resistance detected by Etest and TaqMan real-time polymerase chain reaction: a comparative study. Apmis 120, 712–717. 10.1111/j.1600-0463.2012.02896.x 22882260

[B26] NIH (1994). *Helicobacter pylori* in peptic ulcer disease. Jama 272, 65–69. 10.1001/jama.1994.03520010077036 8007082

[B27] Nishizawa T. Suzuki H. Umezawa A. Muraoka H. Iwasaki E. Masaoka T. (2007). Rapid detection of point mutations conferring resistance to fluoroquinolone in gyrA of *Helicobacter pylori* by allele-specific PCR. J. Clin. Microbiol. 45, 303–305. 10.1128/jcm.01997-06 17122023 PMC1829027

[B28] Nyssen O. P. Bordin D. Tepes B. Pérez-Aisa Á. Vaira D. Caldas M. (2021). European Registry on *Helicobacter pylori* management (Hp-EuReg): patterns and trends in first-line empirical eradication prescription and outcomes of 5 years and 21 533 patients. Gut 70, 40–54. 10.1136/gutjnl-2020-321372 32958544

[B29] Olekhnovich I. N. Goodwin A. Hoffman P. S. (2009). Characterization of the NAD(P)H oxidase and metronidazole reductase activities of the RdxA nitroreductase of *Helicobacter pylori* . Febs J. 276, 3354–3364. 10.1111/j.1742-4658.2009.07060.x 19438716 PMC2751797

[B30] Savoldi A. Carrara E. Graham D. Y. Conti M. Tacconelli E. (2018). Prevalence of antibiotic resistance in *Helicobacter pylori*: a systematic review and meta-analysis in world Health organization regions. Gastroenterology 155, 1372–1382. 10.1053/j.gastro.2018.07.007 29990487 PMC6905086

[B31] Suzuki R. B. Lopes R. A. Da Câmara Lopes G. A. Hung Ho T. Sperança M. A. (2013). Low *Helicobacter pylori* primary resistance to clarithromycin in gastric biopsy specimens from dyspeptic patients of a city in the interior of São Paulo, Brazil. BMC Gastroenterol. 13, 164. 10.1186/1471-230x-13-164 24305035 PMC4235177

[B32] Tomb J. F. White O. Kerlavage A. R. Clayton R. A. Sutton G. G. Fleischmann R. D. (1997). The complete genome sequence of the gastric pathogen *Helicobacter pylori* . Nature 388, 539–547. 10.1038/41483 9252185

[B33] Woo H. Y. Park D. I. Park H. Kim M. K. Kim D. H. Kim I. S. (2009). Dual-priming oligonucleotide-based multiplex PCR for the detection of *Helicobacter pylori* and determination of clarithromycin resistance with gastric biopsy specimens. Helicobacter 14, 22–28. 10.1111/j.1523-5378.2009.00654.x 19191892

[B34] Yang F. Zhang J. Wang S. Sun Z. Zhou J. Li F. (2021). Genomic population structure of *Helicobacter pylori* Shanghai isolates and identification of genomic features uniquely linked with pathogenicity. Virulence 12, 1258–1270. 10.1080/21505594.2021.1920762 33904371 PMC8081043

[B35] Zhang S. Wang X. Wise M. J. He Y. Chen H. Liu A. (2020). Mutations of *Helicobacter pylori* RdxA are mainly related to the phylogenetic origin of the strain and not to metronidazole resistance. J. Antimicrob. Chemother. 75, 3152–3155. 10.1093/jac/dkaa302 32676634

[B36] Zingman L. V. Park S. Olson T. M. Alekseev A. E. Terzic A. (2007). Aminoglycoside-induced translational read-through in disease: overcoming nonsense mutations by pharmacogenetic therapy. Clin. Pharmacol. Ther. 81, 99–103. 10.1038/sj.clpt.6100012 17186006

