# Supplementary material for: Functional determination of site-mutations in rdxA involved in metronidazole resistance of Helicobacter pylori
Source: Front Cell Dev Biol. 2024 Jul 19;12:1435064. doi: 10.3389/fcell.2024.1435064 (PMC11294100; doi:10.3389/fcell.2024.1435064)
Supplement: Supplementary file 1 [file DataSheet1.doc]

**
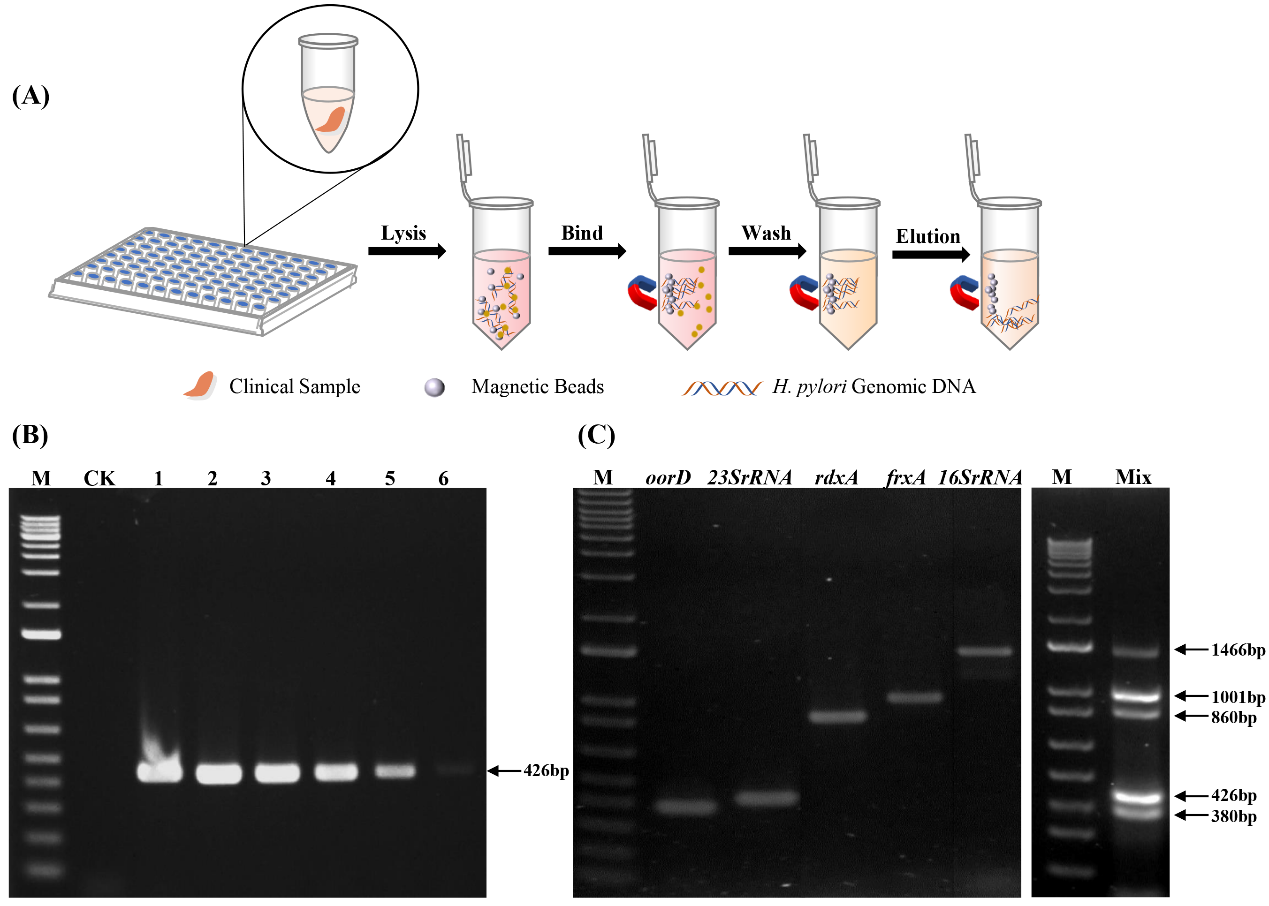
**

**Supplementary Figure 1.** PCR using primers specific for 23S rRNA of *H. pylori*

1. Schematic of DNA extraction. **(B)** Lane CK: *H. pylori* negative sample, Lanes 1-6: *H. pylori* positive sample, genomic DNA was serially diluted 5-fold. **(C)** Multiplex PCR for detection of *oorD, 23S rRNA, rdxA, frxA, 16S rRNA*.

**
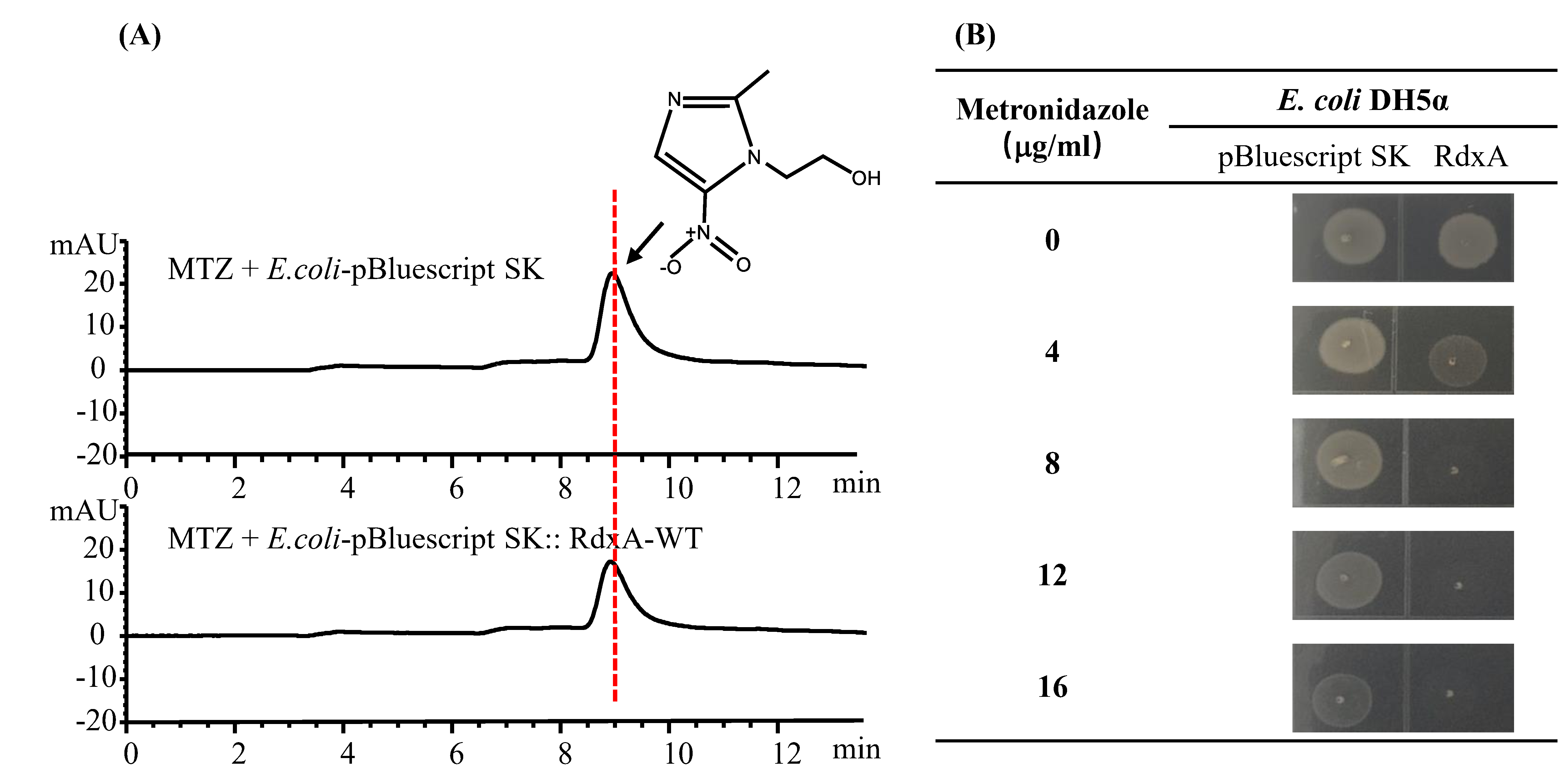
**

**Supplementary Figure 2.** *E. coli* DH5α was used as heterologous host for RdxA to characterize MTZ resistance

(A) HPLC chromatogram of *E. coli* DH5α liquid medium containing MTZ, the MTZ content (16 μg/ml) in *E. coli* liquid medium expressing RdxA (below) was reduced by 24% compared with the control group (above). (B) *E. coli* DH5α transformed with the empty vector pBluescript SK showed inherent MTZ resistance, while the *E. coli* DH5α transformed with wild type RdxA (from strain 26695) acquired a metronidazole-sensitive phenotype.

**Supplementary Table 1. Primers used in** this study

| **Primer** | **Sequence** | **Product Size** | **Use** |
| --- | --- | --- | --- |
| *23S rRNA*-F | ACTAGCACCGTAAGTTCGC | 426bp | Validation of the H. pylori genome |
| *23S rRNA*-R | AGCCTCCCACCTATCCTG |
| *oorD*-F | CAAAGGAGAATGAATGGCTAAAATGAG | 380bp |
| *oorD*-R | TTCCCATCAGAAATAATCTCACGCA |
| *frxA*-F | GCCCGTGCATAGGCGTATTTTG | 1001bp |
| *frxA*-R | GCGCTCTGGCCTTAGCCTTTTCTA |
| *16S rRNA*-F | CGGTTACCTTGTTACGACTTCAC | 1466bp |
| *16S rRNA*-R | TATGGAGAGTTTGATCCTG |
| *rdxA*-F1 | AGCATGGGGCAGATTTTAAG | 860bp | verification and Construction of the pBluescrip *rdxA* |
| *rdxA*-R1 | CTCTTAGCGCTTAATGAAACGC |
| *rdxA*-F2 | TCGAATTCAGCATGGGGCAGATTT | 877bp | Construction of the pBluescrip *rdxA* |
| *rdxA*-R2 | CGTGGATCCCTCTTAGCGCTTAATG |
| R16H-F | AACGAGCACCATTCTTGCAAGA | 3806bp | Construction of Mutants |
| R16H-R | AGAATGGTGCTCGTTTAATAAT |
| R16C-F | AAACGAGTGCCATTCTTGCAAG |
| R16C-R | GAATGGCACTCGTTTAATAATT |
| S18F-F | CGCCATTTTTGCAAGATGTTTG |
| S18F-R | CTTGCAAAAATGGCGCTCGTTT |
| S29T-F | TGAGTTTACTAGCACAGAATTAG |
| S29T-R | GTGCTAGTAAACTCATAATGGC |
| S30G-F | GTTTTCTGGCACAGAATTAGAAG |
| S30G-R | TCTGTGCCAGAAAACTCATAATG |
| S43L-F | CCAGGCTATTGCCAAGCTCTTACAAC |
| S43L-R | GAGCTTGGCAATAGCCTGGCGATTTCA |
| D59S-F | ATGGTTACTAGTAAGGATTTAAAAAAA |
| D59S-R | AAATCCTTACTAGTAACCATCACAAA |
| L62I-F | GATAAGGATATAAAAAAACAAATTGCA |
| L62I-R | TTGTTTTTTTATATCCTTATCAGTAA |
| S79N-F | TGATTAAAAACGCTTCAGCGTTAATGG |
| S79N-R | CGCTGAAGCGTTTTTAATCATCTCTTC |
| A82V-F | AAGCGCTTCAGTGTTAATGGTGGTATGCTCTTTAAG |
| A82V-R | ACCACCATTAACACTGAAGCGCTTTTAATCATCTCT |
| S92N-F | AGACCCAACGAGTTGTTACCACA |
| S92N-R | CAACTCGTTGGGTCTTAAAGAGC |
| Q146R-F | TGTGGGGCGAATTTGCATGGGCGT |
| Q146R-R | TGCAAATTCGCCCCACAGCGATAT |
| E174K-F | AGTTTTAAAAGAGCGTATCAAT |
| E174K-R | CGCTCTTTTAAAACTTCGCCCA |
| P180S-F | CAATAAGTCTAAAATCGCATGCT |
| P180S-R | ATTTTAGACTTATTGATACGCT |
| A187V | CATGCTTGATCGTATTGGGCAAGAGGGTGGCAGAAG |
| A187V | CTCTTGCCCAATACGATCAAGCATGCGATTTTAGGC |
| A193V | GCAAGAGGGTGGTAGAAGCGAGTCAAAAATCAAGAA |
| A193V | TGACTCGCTTCTACCACCCTCTTGCCCAAAGCGATC |

**Supplementary Table 2.** RdxA protein information of 57 reference strains

| **Accession** | **Strain** | **Population type** | **Assembly** |
| --- | --- | --- | --- |
| WP_120860235.1 | HP00152 | hpEurope | ASM363862v1 |
| WP_120993484.1 | HP01306 | hpEurope | ASM363858v1 |
| WP_120860235.1 | HP03054 | hpEurope | ASM363824v1 |
| WP_033621559.1 | HP04087 | hpEurope | ASM363819v1 |
| WP_120839699.1 | HP11005 | hpEurope | ASM363774v1 |
| WP_120940322.1 | HP13027 | hpEurope | ASM363720v1 |
| WP_000670110.1 | HP26695 | hpEurope | ASM30779v1 |
| WP_120860235.1 | HPJ040 | hpEurope | ASM363555v1 |
| WP_120866167.1 | HPJ099 | hpEurope | ASM363620v1 |
| WP_120860235.1 | HPJ118 | hpEurope | ASM363621v1 |
| WP_000669987.1 | HP01102 | hpAfrica1 | ASM363829v1 |
| WP_120861648.1 | HP06059 | hpAfrica1 | ASM363788v1 |
| WP_121275951.1 | HP08072 | hpAfrica1 | ASM363782v1 |
| WP_000669987.1 | HP09046 | hpAfrica1 | ASM363775v1 |
| WP_120892162.1 | HP12020 | hpAfrica1 | ASM363834v1 |
| WP_000669987.1 | HP13011 | hpAfrica1 | ASM363737v1 |
| WP_120900540.1 | HP13026 | hpAfrica1 | ASM363722v1 |
| WP_120826287.1 | HP99216 | hpAfrica1 | ASM363571v1 |
| WP_120826287.1 | HP99330 | hpAfrica1 | ASM363566v1 |
| WP_120885126.1 | HP99648 | hpAfrica1 | ASM363632v1 |
| WP_120820665.1 | HP12054 | hpAfrica2 | ASM363762v1 |
| WP_121036244.1 | HP12073 | hpAfrica2 | ASM363748v1 |
| WP_000670061.1 | SouthAfrica7 | hpAfrica2 | ASM18524v1 |
| WP_121095336.1 | HP15054 | hpAsia2 | [ASM363654v1](https://www.ncbi.nlm.nih.gov/assembly/2012071) |
| WP_121070284.1 | HP99244 | hpAsia2 | [ASM363635v1](https://www.ncbi.nlm.nih.gov/assembly/2011971) |
| WP_121018436.1 | HP12036 | hpEastAsia | ASM363798v1 |
| WP_120878472.1 | HP12069 | hpEastAsia | ASM363751v1 |
| WP_120836615.1 | HP12078 | hpEastAsia | ASM363743v1 |
| WP_120881881.1 | HP13063 | hpEastAsia | ASM363702v1 |
| WP_120801243.1 | HP14031 | hpEastAsia | ASM363690v1 |
| WP_120814716.1 | HP14051 | hpEastAsia | ASM363680v1 |
| WP_121293391.1 | HP14069 | hpEastAsia | ASM363670v1 |
| WP_120902311.1 | HP15003 | hpEastAsia | ASM363666v1 |
| WP_120934889.1 | HP15004 | hpEastAsia | ASM363664v1 |
| WP_121050015.1 | HP15018 | hpEastAsia | ASM363606v1 |
| WP_121297193.1 | HP15020 | hpEastAsia | ASM363660v1 |
| WP_120847002.1 | HP15027 | hpEastAsia | ASM363600v1 |
| WP_120931844.1 | HP15036 | hpEastAsia | ASM363588v1 |
| WP_120937842.1 | HP15040 | hpEastAsia | ASM363655v1 |
| WP_000670095.1 | HP15051 | hpEastAsia | ASM363650v1 |
| WP_121011792.1 | HP01234 | hpNEAfrica | ASM363859v1 |
| WP_121092370.1 | HP07036 | hpNEAfrica | ASM363787v1 |
| WP_121105762.1 | HP08074 | hpNEAfrica | ASM363777v1 |
| WP_120869170.1 | HP11049 | hpNEAfrica | ASM363804v1 |
| WP_120832144.1 | HP13005 | hpNEAfrica | ASM363741v1 |
| WP_120906507.1 | HP13050 | hpNEAfrica | ASM363711v1 |
| WP_120908454.1 | HP13068 | hpNEAfrica | ASM363698v1 |
| WP_121054435.1 | HP14039 | hpNEAfrica | ASM363685v2 |
| WP_120934673.1 | HP15005 | hpNEAfrica | ASM363616v1 |
| WP_121288910.1 | HP98123 | hpNEAfrica | ASM363639v1 |
| WP_120911143.1 | HP98490 | hpNEAfrica | ASM363638v1 |
| WP_120884546.1 | HP99255 | hpNEAfrica | ASM363570v1 |
| - | HP01324 | hpSahul | ASM830448v1 |
| - | HPJ071 | hpSahul | ASM830424v1 |
| - | HPJ148 | hpSahul | ASM830419v1 |
| - | HPJ156 | hpSahul | ASM830417v1 |
| - | HPJ207 | hpSahul | ASM830422v1 |

“-” means the homologous sequence of RdxA of this strain has no accession number.

**Supplementary Table 3. Alignment of full-length RdxA sequences derived from 54 *H. pylori*-positive samples and reference strains belonging to hpEurope, hpNEAfrica and hpEastAsia**

| **Strains and samples** | **Mutations** | | | | | | | | | | | | | | | | | | | | | | | | | | | | | | | | | | | | | | | | | | | | | | | | |
| --- | --- | --- | --- | --- | --- | --- | --- | --- | --- | --- | --- | --- | --- | --- | --- | --- | --- | --- | --- | --- | --- | --- | --- | --- | --- | --- | --- | --- | --- | --- | --- | --- | --- | --- | --- | --- | --- | --- | --- | --- | --- | --- | --- | --- | --- | --- | --- | --- | --- |
|  | **5** | **6** | **16** | **18** | **21** | **25** | **29** | **30** | **31** | **43** | **47** | **53** | **54** | **56** | **59** | **62** | **64** | **68** | **74** | **79** | **80** | **82** | **88** | **90** | **91** | **92** | **96** | **97** | **98** | **108** | **116** | **117** | **118** | **131** | **134** | **135** | **136** | **146** | **163** | **165** | **172** | **173** | **174** | **180** | **187** | **193** | **204** | **205** | **206** |
| HP26695 hpEurope | D | Q | R | S | M | H | S | S | T | S | Y | H | F | M | D | L | K | A | E | S | A | A | S | R | P | S | P | H | G | S | S | F | A | R | S | Y | - | Q | G | D | V | L | E | P | A | A | V | D | A |
| HPJ118 hpEurope |  |  |  |  |  |  |  |  | E |  |  |  |  | I | N |  |  |  |  |  |  |  |  | K |  |  |  |  | S |  |  |  |  |  |  |  | - |  |  |  |  |  |  |  |  |  |  |  |  |
| HPJ099 hpEurope |  |  |  |  |  |  |  |  | E |  |  |  |  |  | N |  |  |  |  |  |  |  |  | K |  |  |  |  |  |  |  |  | S |  |  |  | - |  |  |  |  |  |  |  |  |  |  |  |  |
| HPJ040 hpEurope |  |  |  |  |  |  |  |  | E |  |  |  |  | I | N |  |  |  |  |  |  |  |  | K |  |  |  |  | S |  |  |  |  |  |  |  | - |  |  |  |  |  |  |  |  |  |  |  |  |
| HP13027 hpEurope |  |  |  |  |  |  |  |  | E |  |  |  |  |  | N |  |  | V |  |  |  |  |  |  |  |  |  | T |  |  |  |  |  |  |  |  | - |  |  |  |  |  |  |  |  |  |  |  |  |
| HP11005 hpEurope |  |  | H |  |  |  |  |  | E |  |  |  |  |  | N |  |  | V |  |  |  |  |  | K |  |  |  |  | S | T |  |  | S |  |  |  | - |  |  |  |  |  |  |  |  |  |  |  |  |
| HP04087 hpEurope |  |  |  |  |  |  |  |  | E |  |  |  |  |  | N |  |  |  |  |  |  |  |  |  |  |  |  |  |  |  |  |  |  |  |  |  | - |  |  |  |  |  |  |  |  |  |  |  |  |
| HP03054 hpEurope |  |  |  |  |  |  |  |  | E |  |  |  |  | I | N |  |  |  |  |  |  |  |  | K |  |  |  |  | S |  |  |  |  |  |  |  | - |  |  |  |  |  |  |  |  |  |  |  |  |
| HP01306 hpEurope |  |  |  |  |  | Y |  |  | E |  |  |  |  | I | N |  |  |  |  |  |  |  |  | K |  |  |  |  | S |  |  |  | T |  |  |  | - |  |  |  | I |  |  |  |  |  |  |  |  |
| HP00152 hpEurope |  |  |  |  |  |  |  |  | E |  |  |  |  | I | N |  |  |  |  |  |  |  |  | K |  |  |  |  | S |  |  |  |  |  |  |  | - |  |  |  |  |  |  |  |  |  |  |  |  |
| HPSH010 PP950257 |  |  |  |  |  |  |  |  |  |  |  |  |  |  |  |  |  |  |  |  |  |  |  |  |  |  |  |  |  |  |  |  |  |  |  |  | - |  |  |  |  |  | K |  |  |  |  |  |  |
| HPSH012 PP950259 |  |  |  |  |  |  |  |  | E |  |  |  |  |  |  |  |  |  |  |  |  |  |  |  |  |  |  |  |  |  |  |  |  |  |  |  | - |  |  |  |  |  |  |  |  |  |  |  |  |
| HPSH014 PP950261 |  |  |  |  |  |  |  |  |  |  |  |  |  |  |  |  |  |  |  |  |  |  |  |  |  |  |  |  |  |  |  |  |  |  |  |  | - |  |  |  |  |  | K |  |  |  |  |  |  |
| HP99255 hpNEAfrica |  | H |  |  |  |  |  |  |  |  |  |  |  |  | N |  |  |  |  |  |  |  |  | K |  |  |  |  | S |  |  |  | T | K |  |  | - |  |  |  | I |  |  |  |  |  | I |  |  |
| HP98490 hpNEAfrica |  | H |  |  |  |  |  |  |  |  |  |  |  |  | N |  |  | T |  |  |  |  |  | K |  |  |  |  | S |  |  |  | S | K |  |  | - |  |  |  |  |  |  |  |  |  | I |  |  |
| HP98123 hpNEAfrica |  | H |  |  |  |  |  | N | E |  |  |  |  |  | N |  |  |  |  |  |  |  |  |  |  |  |  |  | S |  |  |  | T | K |  |  | - |  |  |  | I |  |  |  |  |  |  |  |  |
| HP15005 hpNEAfrica |  | H | C |  |  |  |  |  | E |  |  |  |  |  | N |  |  |  |  |  |  |  |  | K |  |  |  |  | S |  |  |  | S | K |  |  | - |  |  |  | I |  |  |  |  |  |  |  |  |
| HP14039 hpNEAfrica |  | H |  |  |  |  |  |  | E | L |  |  |  |  | N |  | N |  |  |  |  |  |  | K |  |  |  |  |  |  |  |  |  | K |  |  | - |  |  |  |  |  |  |  |  |  |  |  |  |
| HP13068 hpNEAfrica |  | H |  |  |  |  |  | N |  |  |  |  |  |  | N |  |  |  |  |  |  |  |  | K |  |  |  | T |  |  |  |  |  | K |  |  | - |  |  |  | I |  |  |  |  |  | I |  |  |
| HP13050 hpNEAfrica |  | H |  |  |  |  |  |  |  |  |  |  |  |  | N |  |  |  |  |  |  |  |  | K |  |  |  |  | S |  |  |  |  | K |  |  | - |  |  |  | I |  |  |  |  |  | I |  |  |
| HP13005 hpNEAfrica |  | H |  |  |  |  |  |  |  |  |  |  |  |  | N |  |  |  |  |  |  |  |  | K |  |  |  |  | S |  |  |  |  | K |  |  | - |  |  |  | I |  |  |  |  |  | I |  |  |
| HP11049 hpNEAfrica |  | H | H |  |  |  |  |  |  |  |  |  |  |  | N |  |  |  |  |  |  |  |  | K |  |  |  |  | S |  |  |  |  | K |  |  | - |  |  |  | I |  |  |  |  |  | I |  |  |
| HP08074 hpNEAfrica |  | H | C |  |  |  |  |  |  |  |  |  |  |  | N |  |  |  |  |  |  |  |  | K |  |  |  |  | S |  |  |  | S | K |  |  | - |  |  |  | I |  |  |  |  |  | I |  |  |
| HP07036 hpNEAfrica |  | H |  |  |  |  |  |  |  |  |  |  |  |  | N |  |  |  |  |  |  |  |  | K |  |  |  | T |  |  |  |  | T | K |  |  | - |  |  |  |  |  |  |  |  |  |  |  |  |
| HP01234 hpNEAfrica |  | H | H |  |  |  |  |  |  |  |  |  |  |  | N |  |  | V |  |  |  |  |  | K |  |  |  |  | S |  |  |  | T | K |  |  | - |  |  |  | I |  |  |  |  |  |  |  |  |
| HPSH016 PP950263 |  | H |  |  |  |  |  |  |  |  |  |  |  |  | N |  |  |  |  |  |  |  |  | K |  |  |  |  |  |  |  |  | T | K |  |  | - |  |  |  |  |  |  |  |  |  |  |  |  |
| HPSH049 PP950296 |  | H | C |  |  |  |  |  |  |  |  |  |  |  | N |  |  |  |  |  | T |  |  |  |  |  |  | T |  | A |  |  | S | K |  |  | - |  |  |  | I |  |  |  |  |  | I |  |  |
| HP15051 hpEastAsia |  |  |  |  |  |  |  |  | E |  |  | R |  |  | N | V |  |  |  |  |  |  | P |  |  |  |  |  | S |  |  |  |  | K |  |  | - |  |  |  | I |  |  |  |  |  |  |  |  |
| HP15040 hpEastAsia |  |  |  |  | A |  |  |  | E |  |  | R |  |  | N | V |  |  |  |  |  |  | P |  |  |  |  |  | S |  |  |  |  | K |  |  | - |  |  |  | I |  |  |  |  |  |  |  |  |
| HP15036 hpEastAsia |  |  |  |  |  |  |  |  | E |  |  | R |  |  | N | V |  |  |  |  |  |  | P |  |  |  |  |  | S |  |  |  | S | K |  |  | - |  |  |  | I |  |  |  |  |  |  |  | T |
| HP15027 hpEastAsia |  |  | C |  |  |  |  |  | E |  |  |  |  |  | N | V |  |  |  |  |  |  | P |  |  |  |  |  | S |  |  |  |  | K |  |  | - |  |  |  | I |  |  |  |  |  |  |  |  |
| HP15020 hpEastAsia |  |  | H |  |  |  |  |  | E |  |  | R |  |  | N | V |  |  |  |  |  |  | P |  |  |  |  |  | S |  |  |  | T | K |  |  | - |  |  |  | I |  |  |  |  |  |  |  |  |
| HP15018 hpEastAsia |  |  | C |  |  |  |  |  | E |  |  | R |  |  | N | V |  |  |  |  |  |  | P |  |  |  |  |  | S |  |  |  |  | K |  |  | - |  |  |  | I |  |  |  |  |  |  |  | T |
| HP15004 hpEastAsia |  |  | H |  |  |  |  |  | E |  |  |  |  |  | N | V |  |  |  |  |  |  | P |  |  |  |  |  | S |  |  |  |  | K |  |  | - |  |  |  | I |  |  |  |  |  |  |  |  |
| HP15003 hpEastAsia |  |  |  |  |  |  |  |  | E |  |  | R |  |  | N | V |  |  |  |  |  |  | P |  |  |  |  |  | N |  |  |  | S | K |  |  | - |  |  |  | I |  |  |  |  |  |  |  |  |
| HP14069 hpEastAsia |  |  |  |  |  |  |  |  | E |  |  | R |  |  | N |  |  |  |  |  |  |  | P |  |  |  |  |  | S |  |  |  |  | K |  |  | - |  |  |  |  |  |  |  |  |  |  |  |  |
| HP14051 hpEastAsia | N |  |  |  |  |  |  |  | E |  |  | R |  |  | N | I |  |  |  |  |  |  | P |  |  |  |  |  | S |  |  |  |  | K |  |  | - |  |  |  | I |  |  |  |  |  |  |  | T |
| HP14031 hpEastAsia |  |  |  |  |  |  |  |  |  |  |  | R |  |  | N | V |  |  |  |  |  |  | P |  |  |  |  |  | S |  |  |  |  | K |  |  | - |  |  |  | I |  |  |  |  |  |  |  | T |
| HP13063 hpEastAsia |  |  |  |  |  |  |  |  | E |  |  | R |  |  | N | V |  | V |  |  |  |  | P |  |  |  |  |  | S |  |  |  |  | K |  |  | - |  |  |  | I |  |  |  |  |  | I | A |  |
| HP12078 hpEastAsia |  |  |  |  |  |  |  |  | E |  |  | R |  |  | N | V |  |  |  |  |  |  | P |  |  |  |  |  | S |  |  |  |  | K |  |  | - |  |  |  | I |  |  |  |  |  |  | A |  |
| HP12069 hpEastAsia |  |  |  |  |  |  |  |  | E |  |  | R |  |  | N | V |  |  |  |  |  |  | P |  |  |  |  |  | S |  |  |  |  | K |  |  | - |  |  |  | I |  |  |  |  |  |  | A |  |
| HP12036 hpEastAsia |  |  |  |  |  |  |  |  | E | L |  |  |  |  | N |  |  |  |  |  |  |  | P |  |  |  |  |  | S |  |  |  |  | K |  |  | - |  |  |  | I |  |  |  |  |  |  |  |  |
| HPSH001 PP950248 |  |  |  |  |  |  |  |  | E |  |  |  |  |  | N | V |  |  |  |  |  |  | P |  |  |  |  |  | S |  |  |  | S | K |  |  | - |  |  |  | I |  |  | S |  |  |  |  | T |
| HPSH002 PP950249 |  |  |  |  |  |  |  |  | E | L |  | R |  |  | N |  |  |  |  |  |  |  | P |  |  |  |  |  | S |  | F |  |  | K |  |  | - |  |  |  | I |  |  |  |  |  |  |  |  |
| HPSH003 PP950250 |  |  |  |  |  |  | T |  | E |  |  | R |  |  | N |  |  |  |  |  |  |  | P |  |  |  |  |  | S |  |  |  | S | K |  |  | - |  |  |  | I |  |  |  |  |  |  |  | T |
| HPSH004 PP950251 |  |  |  |  |  |  |  |  | E |  |  |  |  |  | N | V |  |  |  |  |  |  | P |  |  |  |  |  | S | A |  |  |  | K |  | S | - |  |  |  | I |  |  |  |  |  |  |  |  |
| HPSH005 PP950252 | N |  |  |  |  |  |  |  | E |  |  | R |  |  | N | V |  |  |  |  |  |  | P |  |  |  |  |  | S |  |  |  |  | K | N |  | - |  |  |  | I |  |  |  |  |  |  |  |  |
| HPSH006 PP950253 |  |  |  |  |  |  |  |  | E |  |  | R |  |  | N | V |  |  |  |  |  |  | P |  |  |  |  |  | S |  |  |  |  | K |  |  | - |  |  |  | I |  |  |  |  |  |  |  | T |
| HPSH007 PP950254 |  |  |  |  |  |  |  |  | E |  |  | R |  |  | N |  |  |  |  |  |  |  | P |  |  |  |  |  | S |  |  |  |  | K |  |  | - |  |  |  | I |  |  |  |  |  |  |  | T |
| HPSH008 PP950255 |  |  |  |  |  |  |  |  | E |  |  | R |  |  | N |  |  |  |  |  |  |  | P |  |  |  |  |  | S |  |  |  |  | K |  |  | - |  |  |  | I |  |  |  |  |  |  |  | T |
| HPSH009 PP950256 |  |  |  |  | I |  |  |  | E |  |  | R |  |  | N | I |  |  |  |  |  |  | P |  | S |  |  |  | S |  |  |  |  | K |  |  | - |  |  |  | I |  |  |  |  | V |  |  |  |
| HPSH011 PP950258 |  |  | H |  | A |  |  |  | E |  |  | R |  |  | N | V |  |  |  |  |  |  | P |  | S |  |  |  | S |  |  |  |  | K |  |  | - |  |  |  | I |  |  |  |  |  |  |  | T |
| HPSH013 PP950260 |  |  |  |  |  |  |  |  |  |  |  | R |  |  | N | V |  |  |  |  |  |  | P |  |  |  |  |  | S |  |  |  | S | K |  |  | - |  |  |  | I |  |  |  |  |  |  |  |  |
| HPSH015 PP950262 |  |  |  |  |  |  |  |  | E |  |  | R |  |  | N | V |  |  |  |  |  |  | P |  |  |  |  |  | S |  |  |  |  | K |  |  | - |  |  |  | I |  |  |  |  |  |  |  | T |
| HPSH017 PP950264 |  |  |  |  |  |  |  |  | E |  |  | R |  |  | N | V |  | V |  |  |  |  | P |  |  |  |  |  | S |  |  |  |  | K |  |  | - |  |  |  | I |  |  |  |  |  |  |  | T |
| HPSH018 PP950265 |  |  |  |  |  |  |  |  | E |  |  | R |  |  | N | V |  |  |  |  |  |  | P |  |  |  |  |  | S | A |  |  |  | K |  |  | - |  | V |  | I |  |  |  |  |  |  |  |  |
| HPSH019 PP950266 |  |  |  |  |  |  |  | G | E |  |  | R |  |  | N | V |  |  |  | N |  |  | P |  | S |  |  |  | S |  |  |  |  | K |  |  | - |  |  |  | I |  |  |  |  |  |  |  |  |
| HPSH020 PP950267 |  |  |  |  |  |  |  |  | E |  |  | R |  |  | N | V |  |  |  |  |  |  | P |  |  |  |  |  | S |  |  |  |  | K |  |  | - |  |  |  | I |  |  |  |  |  |  |  | T |
| HPSH021 PP950268 |  |  |  |  |  |  |  |  | E |  |  | R |  |  | N | V |  |  |  |  |  |  | P |  |  |  |  |  | S |  |  |  | S | K |  |  | - |  |  |  | I |  |  |  |  |  |  |  | T |
| HPSH022 PP950269 |  |  |  |  | A |  |  |  | E |  |  | R |  |  | N |  | N |  |  |  |  |  | P |  |  |  |  |  | S |  |  |  |  | K |  |  | - |  |  |  | I |  |  |  |  |  |  |  | T |
| HPSH023 PP950270 |  |  |  |  |  |  |  |  | E |  |  |  |  |  | N | V |  |  |  |  |  |  | P |  |  |  | L |  | S | A |  |  | S | K |  |  | - |  |  |  |  |  |  |  |  |  | I |  |  |
| HPSH024 PP950271 | N |  |  |  |  |  |  |  | E |  |  | R |  |  | N | V |  |  |  |  |  |  | P |  |  |  |  |  | S |  |  |  | S | K |  |  | - |  |  |  | I |  |  |  |  |  |  |  |  |
| HPSH025 PP950272 | N |  |  |  |  |  |  |  | E |  |  | R |  | I | N | V |  |  |  |  |  |  | P |  |  |  |  |  | S |  |  |  |  | K |  |  | - |  |  |  | I |  |  |  |  |  |  |  |  |
| HPSH026 PP950273 | N |  |  |  |  |  |  |  | E |  |  | R |  |  | N | V |  |  |  |  |  |  | P |  |  |  |  |  | S |  |  |  | S | K |  |  | - |  |  |  | I |  |  |  |  |  |  |  |  |
| HPSH027 PP950274 | N |  |  |  |  |  |  |  | E |  |  | R |  |  | N | V |  |  |  |  |  |  | P |  |  |  |  |  | S |  |  |  | S | K |  |  | - |  |  |  | I |  |  |  |  |  |  |  |  |
| HPSH028 PP950275 | N |  |  |  |  |  |  |  | E |  |  | R |  |  | N | V |  |  |  |  |  |  | P |  |  |  |  |  | S |  |  |  | S | K |  |  | - |  |  |  | I |  |  |  |  |  |  |  |  |
| HPSH029 PP950276 |  |  |  |  |  |  |  |  | E |  |  | R |  |  | N | V |  |  |  |  |  |  | P |  |  |  |  |  | S |  |  |  |  | K |  |  | - |  |  |  | I |  |  |  |  |  |  |  |  |
| HPSH030 PP950277 |  |  |  |  |  |  |  |  | E |  |  | R |  |  | N | V |  |  |  |  |  |  | P |  |  |  |  |  | S |  |  |  |  | K |  |  | - |  |  |  | I |  |  |  |  |  |  |  | T |
| HPSH031 PP950278 |  |  |  |  |  |  |  |  | E |  |  | R |  |  | S |  |  |  |  |  |  |  | P |  |  | N |  | Y | S |  |  |  |  | K |  |  | - |  |  |  | I |  |  |  |  |  |  |  |  |
| HPSH032 PP950279 |  |  |  |  |  |  |  |  | E |  |  | R |  |  | N | V |  |  |  |  |  |  | P |  |  |  |  |  | S |  |  |  |  | K |  |  | - |  |  |  | I |  |  |  |  |  |  |  |  |
| HPSH033 PP950280 |  |  |  |  | A |  |  |  | E |  |  | R |  |  | N | V |  |  |  |  |  |  | P |  |  |  |  |  | S |  |  |  |  | K |  |  | - |  |  |  | I |  |  |  |  |  |  |  | T |
| HPSH034 PP950281 |  |  | H |  |  |  |  |  | E |  | C | R |  |  | N | V |  |  |  |  |  |  | P |  |  |  |  |  | S |  |  |  |  | K |  |  | - |  |  |  | I |  |  |  |  |  |  |  | T |
| HPSH035 PP950282 |  |  |  |  |  |  |  |  | E |  |  | R |  |  | S |  |  |  |  |  |  |  | P |  |  | N |  | Y | S |  |  |  |  | K |  |  | - |  | V |  | I |  |  |  |  |  |  |  |  |
| HPSH036 PP950283 |  |  |  |  | A |  |  |  | E |  |  | R |  |  | N | V |  |  |  |  |  |  | P |  |  |  |  |  | S |  |  |  |  | K |  |  | - |  |  |  | I |  |  |  |  |  |  |  |  |
| HPSH037 PP950284 |  |  |  |  |  |  |  |  | E |  |  | R |  |  | N | V |  |  |  |  |  |  | P |  |  |  |  |  | S | A |  |  |  | K |  |  | - |  | V |  | I |  |  |  |  |  |  |  |  |
| HPSH038 PP950285 |  |  | C |  | A |  |  |  | E |  |  | R |  |  | N | V |  |  |  |  |  | V | P |  |  |  |  |  | S |  |  |  |  | K |  |  | - |  |  |  | I |  |  |  |  |  |  |  | T |
| HPSH039 PP950286 |  |  |  |  |  |  |  |  | E |  |  | R |  |  | N | V |  | T |  |  |  |  | P |  |  |  |  |  | S |  |  |  | S | K |  |  | - |  |  |  | I |  |  |  |  |  |  |  | T |
| HPSH040 PP950287 |  |  |  |  |  |  |  |  | E |  |  | R |  |  | N | V |  | T |  |  |  |  | P |  |  |  |  |  | S |  |  |  |  | K |  |  | - |  |  |  | I |  |  |  | V |  |  |  | T |
| HPSH041 PP950288 |  |  | H |  |  |  |  |  | E |  |  | R |  |  | N | V |  |  | G |  |  |  | P |  |  |  |  |  | S |  |  |  |  | K |  |  | - |  |  |  | I |  |  |  |  |  |  |  | T |
| HPSH042 PP950289 |  |  |  |  |  |  |  |  | E |  |  | R |  |  | N |  |  |  |  |  |  |  | P |  | S |  |  |  | S |  | F |  |  | K |  |  | - |  |  |  | I |  |  |  |  |  |  |  |  |
| HPSH043 PP950290 |  |  |  | F |  |  |  |  | E |  |  | R |  |  | N |  |  | V |  |  |  |  | P |  | S |  |  |  | S |  |  |  |  | K |  |  | - |  |  |  | I |  |  |  |  |  |  |  |  |
| HPSH044 PP950291 |  |  | H |  | A |  |  |  | E |  |  | R |  |  | N | V |  |  |  |  |  |  | P |  |  |  |  |  | S |  |  |  |  | K |  |  | - |  |  |  | I |  |  | S |  |  | I |  | T |
| HPSH045 PP950292 |  |  | H |  | A | Y |  |  | E |  |  | R |  |  | N |  |  |  |  |  | T |  | P |  |  |  |  |  | S |  |  |  |  | K |  |  | - |  |  |  | I |  |  |  |  |  |  |  |  |
| HPSH046 PP950293 |  |  | H |  |  | R |  |  | E |  |  | R |  |  | N | V |  |  |  |  |  |  | P |  |  |  |  |  | S | A |  |  |  | K |  |  | - |  |  |  | I |  |  |  |  |  |  |  |  |
| HPSH047 PP950294 |  |  |  |  |  |  |  |  | E |  |  | R |  |  | N | V |  |  |  |  |  |  | P |  |  |  |  |  | S |  |  |  |  | K |  |  | I |  |  |  | I |  |  |  |  |  | I |  | T |
| HPSH048 PP950295 |  |  | C |  |  |  |  |  | E |  |  | R |  |  | S | V |  |  |  |  |  |  | P |  |  |  |  |  | S |  |  |  |  | K |  |  | - | R |  |  | I |  |  |  |  |  |  |  | T |
| HPSH050 PP950297 |  |  | H |  |  | R |  |  | E |  |  | R |  |  | N | V |  |  |  |  |  |  | P |  |  |  |  |  | S | A |  |  |  | K |  |  | - |  |  |  | I |  |  |  |  |  |  |  |  |
| HPSH051 PP950298 |  |  | H |  | A |  |  |  | E |  |  |  |  |  | N | V |  |  |  |  |  |  | P |  |  |  |  |  | S |  |  |  |  | K |  |  | - |  |  |  | I |  | K |  |  | T |  |  |  |
| HPSH052 PP950299 |  |  | C |  |  |  |  |  | E |  |  |  |  |  | N |  |  | V |  |  | T |  | P |  |  |  |  |  | S |  |  |  |  | K |  |  | - |  |  |  | I |  |  |  |  | T |  |  | T |
| HPSH053 PP950300 |  |  |  |  |  |  |  |  | E |  |  | R |  |  | N | V |  |  |  |  |  |  | P |  | S |  |  |  | S |  |  |  | S | K |  |  | - |  |  |  | I |  |  |  |  |  |  |  |  |
| HPSH054 PP950301 |  |  |  |  |  |  |  |  | E |  |  | R |  |  | N | V |  | V |  |  |  |  | P |  |  |  |  |  | S |  |  |  |  | K |  |  | - |  |  | - |  |  |  |  |  |  |  |  |  |

The numbers and capital letters in the first two rows indicate the position numbers and amino acid residues of the 26695 strain RdxA protein. Only variable sites from clinical samples are shown, identical amino acid residues are not displayed. Columns highlighted in orange represent conserved amino acid residue acid sites in the RdxA sequence from our strains that may be phylogenetic signals rather than resistance related.
